# Crystal deposition disease of the shoulder at uncommon sites: Diagnostic challenges and nomenclature issues in the absence of synovial fluid analysis

**DOI:** 10.1515/rir-2025-0025

**Published:** 2025-12-27

**Authors:** Angelo Nigro

**Affiliations:** Department of Rheumatology of Lucania- UOSD of Rheumatology, “Madonna delle Grazie” Hospital, Matera, Italy

**Keywords:** calcium pyrophosphate deposition disease, CPPD, shoulder arthritis, chondrocalcinosis, pseudogout, crystal arthropathy

## Abstract

Calcium pyrophosphate deposition (CPPD) disease is a common, age-related crystalline arthropathy with diverse clinical presentations. While the knees and wrists are most frequently affected, shoulder involvement is increasingly recognized, occurring in up to 13% of cases, though often underdiagnosed. This mini-review provides a comprehensive overview of the epidemiology, pathogenesis, clinical manifestations, diagnosis, and treatment of shoulder CPPD, contextualized by an illustrative case of a 78-year-old woman with atypical calcifications in the axillary recess and supraspinatus muscle. A key focus is the diagnostic challenge when synovial fluid analysis, the gold standard for crystal confirmation, is technically unfeasible, a common scenario in clinical practice. We systematically discuss modern imaging techniques (ultrasound, dual-energy computed tomography [CT], conventional radiography) and demonstrate the practical application of the 2023 American College of Rheumatology/European League Against Rheumatism (ACR/EULAR) classification criteria for establishing probable CPPD when crystal analysis is unavailable. The review addresses critical differential diagnosis considerations, particularly distinguishing CPPD from basic calcium phosphate (BCP) deposition disease, and summarizes evidence-based therapeutic strategies for acute pseudogout flares and chronic inflammatory arthritis, including emerging biologic therapies targeting the interleukin-1 (IL-1) pathway. This comprehensive resource aids clinicians managing shoulder calcifications in the absence of definitive crystal confirmation.

## Introduction

Calcium pyrophosphate deposition (CPPD) disease is a metabolic arthropathy characterized by the deposition of calcium pyrophosphate (CPP) crystals in articular and periarticular tissues.^[1]^ Its prevalence increases sharply with age, affecting up to 50% of individuals over 80 years old.^[2]^ The clinical spectrum is broad, ranging from asymptomatic radiographic findings (chondrocalcinosis) to acute, self-limiting inflammatory arthritis (pseudogout), chronic inflammatory polyarthritis mimicking rheumatoid arthritis, and a severe, destructive arthropathy known as “pyrophosphate arthropathy”.^[3]^

The nomenclature surrounding CPPD has been historically confusing. The terms pseudogout, chondrocalcinosis, and pyrophosphate arthropathy have been used interchangeably, leading to ambiguity. In 2011, a European League Against Rheumatism (EULAR) task force recommended restricting “chondrocalcinosis” to a radiographic finding and abandoning “pseudogout” in favor of more precise terms like “acute CPP crystal arthritis” ^[4]^ More recently, the Gout, Hyperuricemia and Crystal-Associated Disease Network (G-CAN) has initiated a project to further standardize this nomenclature.^[5]^

While the knee and wrist are the classic sites of involvement, the shoulder is also a frequent target, although its true prevalence is likely underestimated.^[6]^ Shoulder CPPD can present with acute pain, chronic degenerative changes, or massive rotator cuff tears associated with a destructive glenohumeral arthropathy, a condition sometimes referred to as “Milwaukee shoulder,” which is more classically associated with basic calcium phosphate (BCP) crystals but can coexist with CPPD.^[7,8]^

Diagnosing shoulder CPPD presents unique challenges. Synovial fluid aspiration, the gold standard for crystal identification based on the original criteria by Ryan and McCarty,^[9]^ can be technically difficult in the shoulder joint. Furthermore, imaging findings may overlap with other conditions, most notably BCP deposition disease (calcific tendinitis). The recent development of the 2023 American College of Rheumatology/ European Alliance of Associations for Rheumatology (ACR/EULAR) classification criteria for CPPD disease provides a new framework for standardized case definition, even in the absence of crystal confirmation.^[10]^

This mini-review synthesizes the current understanding of shoulder CPPD, covering its epidemiology, pathogenesis, clinical features, diagnosis, and management, using a complex clinical case to illustrate the diagnostic and therapeutic journey.

A comprehensive literature search was performed using PubMed and MEDLINE databases (January 2000 to October 2025). Search terms included “calcium pyrophosphate deposition disease” OR “CPPD” OR “pseudogout” OR “chondrocalcinosis” combined with “shoulder” OR “glenohumeral” OR “acromioclavicular. “ Articles were included if they reported original data on shoulder CPPD epidemiology, pathogenesis, diagnosis, or treatment. Review articles, case series with ≥5 patients, and landmark historical studies were also included. Non-English articles without available translations were excluded.

## Epidemiology and Pathogenesis

### Epidemiology

The prevalence of radiographic chondrocalcinosis, a surrogate for CPPD, is estimated to be between 7% and 13.7% in adults around 60 years of age, rising to nearly 50% in those older than 80.^[2]^ There is no clear gender predilection, although some studies suggest a slight female predominance after age 75. While knees and wrists are the most commonly affected joints, autopsy and imaging studies have shown that shoulder involvement is common, with CPP deposits found in the glenohumeral joint, acromioclavicular joint, rotator cuff tendons, and surrounding bursae.^[6,11]^ A retrospective study of total joint arthroplasties found pathologically confirmed CPPD in 13% of shoulder specimens, suggesting that shoulder involvement may be more common than clinically recognized.^[12]^

The shoulder represents the third most common site of CPPD involvement after the knee and wrist, with bilateral involvement occurring in approximately 40% of cases. Risk factors for shoulder CPPD include advanced age, osteoarthritis, prior joint trauma, and metabolic disorders such as hyperparathyroidism, hemochromatosis, and hypomagnesemia.

### Pathogenesis

CPPD is fundamentally a disorder of pyrophosphate metabolism. The formation of CPP crystals is driven by an excess of extracellular inorganic pyrophosphate (ePPi) in the cartilage matrix.^[13]^ This process is regulated by a delicate balance of enzymes and transporters:

• **Production of ePPi:** ePPi is generated from Adenosine triphosphate (ATP) by enzymes like ectonucleotide pyrophosphatase/phosphodiesterase 1 (ENPP1). Increased activity of ENPP1 has been observed in CPPD-affected cartilage.

• **Transport of ePPi:** The transmembrane protein (progressive ankylosis protein [ANKH], encoded by the *ANKH* gene) is a key regulator, transporting PPi from the intracellular to the extracellular space. Gain-of-function mutations in *ANKH* are linked to familial forms of CPPD, accounting for approximately 2% of cases.^[14]^

• **Degradation of ePPi:** Tissue-nonspecific alkaline phosphatase (TNAP) hydrolyzes ePPi, acting as a key inhibitor of crystal formation. Reduced TNAP activity has been implicated in CPPD pathogenesis.

An imbalance, with increased ePPi production/transport or decreased degradation, creates a supersaturated environment where CPP crystals precipitate. Aging, osteoarthritis, and metabolic conditions like hemochromatosis, hyperparathyroidism, and hypomagnesemia are major risk factors that disrupt this homeostasis.^[3,13]^ Once formed, CPP crystals trigger a potent inflammatory response by activating the nucleotide-binding oligomerization domain (NOD)-like receptor containing pyrin domain 3 (NLRP3) inflammasome in macrophages, leading to the release of interleukin (IL)-1β and the clinical manifestations of acute arthritis.^[1]^

## Clinical Manifestations and Diagnosis

### Clinical Presentations

Shoulder CPPD can manifest in several ways ^[15]^:

1. **Asymptomatic Chondrocalcinosis:** Incidental finding on imaging, present in up to 30% of elderly patients undergoing shoulder radiography for other reasons.

2. **Acute CPP Crystal Arthritis (Pseudogout):** Sudden onset of severe pain, swelling, warmth, and functional limitation, mimicking an acute rotator cuff tear or septic arthritis. Fever may be present in 50% of cases. Episodes typically last 7–10 days if untreated.

3. **Chronic Pyrophosphate Arthropathy:** A progressive, degenerative process resembling osteoarthritis, often bilateral, with joint space narrowing, subchondral sclerosis, and osteophyte formation. It can lead to a destructive arthropathy with massive rotator cuff tears and glenohumeral instability.^[7,8]^ This presentation may be indistinguishable from primary osteoarthritis without crystal analysis. When severe destructive arthropathy with massive rotator cuff tears is present, the condition overlaps with “Milwaukee shoulder syndrome, “ which was originally described in association with BCP crystals.^[7]^ However, recent studies have shown that CPPD and BCP crystals frequently coexist in these destructive cases, with CPPD found in up to 30% of Milwaukee shoulder specimens. The key clinical distinction is that pure CPPD arthropathy tends to have more prominent inflammatory episodes superimposed on chronic joint damage, while pure BCP disease is typically less inflammatory. Imaging may help differentiate: BCP deposits are usually more dense ( > 400 HU on computed tomography [CT]) and located within tendons, whereas CPP deposits are less dense (150–200 HU) and favor cartilage and synovium.

4. **Tumoral CPPD (Tophaceous Pseudogout):** Rare, large, destructive masses of CPP crystals that can mimic a soft tissue neoplasm or infection.^[16]^

5. **Crowned Dens Syndrome:** Although primarily affecting the atlantoaxial joint, this can present with referred shoulder pain and should be considered in the differential diagnosis.

### An Illustrative Case and Application of ACR/EULAR Criteria

**Figure 1:**
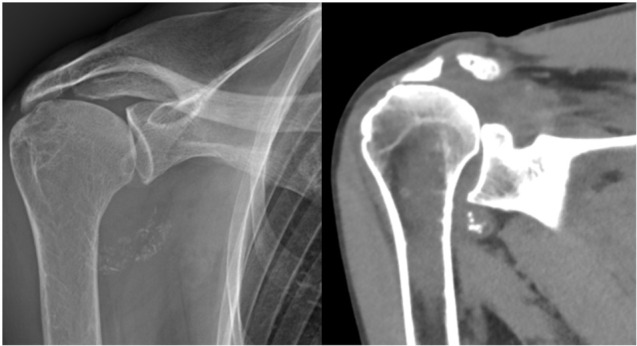
X-ray (left) and computed tomography (CT) scan (right) of the right shoulder showing well-defined calcifications in the axillary recess.

**Figure 2:**
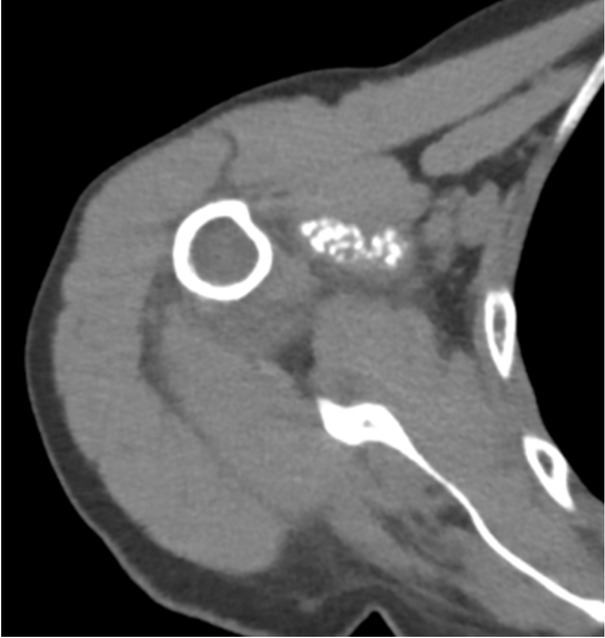
Computed tomography (CT) scan axial view showing the presence of calcifications within the supraspinatus muscle belly, an atypical location for Calcium pyrophosphate deposition (CPPD) deposits.

A 78-year-old woman presented with a one-year history of recurrent, debilitating pain and swelling in her right shoulder, without prior trauma. She reported three distinct episodes of acute pain with complete resolution between episodes. Physical examination revealed tenderness in the axillary recess, pain on abduction beyond 60 degrees, and a positive Neer impingement test. Laboratory tests including erythrocyte sedimentation rate (ESR) (12 mm/h), CRP (0.4 mg/dL), serum calcium, parathyroid hormone, and ferritin were within normal limits. Radiographs and CT scans showed dense, well-defined calcifications in the axillary pouch and within the supraspinatus muscle belly, highly atypical sites for crystal deposition (Figures 1 and 2). Joint aspiration was attempted but was not feasible due to the location of the effusion.

Despite the absence of crystal confirmation, the patient’s presentation was highly suggestive of CPPD. We applied the 2023 ACR/EULAR classification criteria to standardize the diagnosis. The patient met the entry criterion (at least one episode of joint pain, swelling, or tenderness) and had no exclusion criteria. As detailed in Table 1, she achieved a total score of 57, exceeding the classification threshold of > 56.

**Table 1:** Application of 2023 ACR/EULAR classification criteria to the illustrative case

Criterion	Patient Characteristic	Score
Domain 1: Clinical Manifestations		
Level 2: Chronic inflammatory arthritis with acute flares	Chronic pain with acute exacerbations	13
Domain 2: Etiology/Risk Factors		
Level 1: Age > 60 years	78-year-old patient	9
Domain 3: Imaging		
Level 4: Atypical calcifications (periarticular/intramuscular) on CT	Calcifications in axillary recess and supraspinatus muscle	23
Domain 4: Laboratory		
Level 2: Absence of Rheumatoid Factor and anti-CCP	Negative	12
TOTAL SCORE		57
Classification Threshold		> 56

Classification threshold: > 56 points. Entry criterion: At least one episode of joint pain, swelling, or tenderness. Exclusion criteria: None present (trauma with fracture, septic arthritis, tophaceous gout). ACR/EULAR, American College of Rheumatology/European League Against Rheumatism; CT, computed tomography; anti-CCP, Anticitrullinated protein antibodies.

This case highlights the diagnostic challenge of atypical presentations and the utility of the new classification criteria in guiding diagnosis when definitive crystal diagnosis is unobtainable. The calcifications in the axillary recess and intramuscular location within the supraspinatus muscle qualified for the highest imaging score (23 points for “atypical calcifications”) based on the consensus definitions established by Tedeschi *et al*.^[9]^ which specify that periarticular and intramuscular deposits represent atypical features distinct from typical cartilaginous chondrocalcinosis.

### Imaging Features

Imaging plays a crucial role in diagnosing shoulder CPPD, especially when synovial fluid aspiration is challenging.^[17]^

**Plain Radiography:** The most accessible imaging modality, showing chondrocalcinosis as linear or punctate radiodense deposits in hyaline cartilage or fibrocartilage. In the shoulder, calcifications may be seen in the glenohumeral joint cartilage, labrum, or within tendons. Sensitivity is limited (approximately 40%), particularly for early disease.^[18]^

Ultrasound: Increasingly recognized for its diagnostic value in CPPD. CPP deposits appear as hyperechoic deposits within hyaline cartilage, distinct from the superficial location of BCP deposits in calcific tendinitis. The “double contour sign” may be present, though less commonly than in gout. Ultrasound has a sensitivity of 60%–80% and specificity of 90%–95% for detecting CPP deposits when performed by experienced operators.^[19,20]^ Key sonographic features include:-Hyperechoic deposits parallel to the bone surface in hyaline cartilage- Punctate or linear hyperechoic deposits in fibrocartilage- Absence of acoustic shadowing (unlike BCP deposits)

- Dynamic assessment capability during patient movement.

**CT:** Offers superior sensitivity (> 80%) for detecting calcifications and can differentiate CPP from BCP deposits based on attenuation values.^[21]^ CPP deposits typically show attenuation values of 150–200 Hounsfield units, while BCP deposits are typically > 400 HU.^[22]^ CT is particularly valuable for detecting deposits in deep structures like the axillary recess.

**Dual-Energy CT (DECT):** An emerging technique that can differentiate between different types of crystal deposits based on their chemical composition. DECT has shown promising results in distinguishing CPP from BCP and monosodium urate crystals, with sensitivity approaching 90% and specificity > 95%.^[23]^

**Magnetic Resonance Imaging (MRI):** While not ideal for detecting calcifications, MRI is valuable for assessing associated soft tissue changes, synovitis, and ruling out other pathologies. CPP deposits appear as areas of low signal intensity on all sequences. MRI is particularly useful for evaluating rotator cuff integrity and detecting bone marrow edema in chronic cases.

### Differential Diagnosis

Distinguishing shoulder CPPD from other conditions is crucial for appropriate management.

**BCP Deposition Disease:** The most important differential diagnosis. BCP deposits are typically:(1) Located within tendons (especially supraspinatus), (2) More homogeneous and dense on imaging,(3) Associated with acute calcific tendinitis,^[24]^ (4) Show higher attenuation on CT ( > 400 HU), (5) May demonstrate acoustic shadowing on ultrasound

**Septic Arthritis:** Should always be considered in acute presentations. Joint aspiration for cell count and culture is essential when infection is suspected.

**Rheumatoid Arthritis:** Distinguished by symmetric polyarthritis, positive serologies, and characteristic erosions.

**Polymyalgia Rheumatica:** May coexist with CPPD in elderly patients. Distinguished by bilateral shoulder and hip girdle pain with elevated inflammatory markers.

**Frozen Shoulder (Adhesive Capsulitis):** Progressive limitation of passive and active range of motion without significant inflammatory signs.

## Management

Treatment of shoulder CPPD aims to reduce inflammation during acute attacks, prevent recurrent episodes, and manage chronic arthropathy. The approach should be individualized based on presentation severity, comorbidities, and treatment response.

### Acute Flares

The primary goal is rapid resolution of inflammation and restoration of function.

First-line treatment options include:

• **Nonsteroidal anti-inflammatory drugs (NSAIDs):** Effective but should be used with caution in elderly patients with comorbidities. Indomethacin 50 mg three times daily or naproxen 500 mg twice daily for 7–10 days are commonly used regimens.

• **Colchicine:** Low-dose colchicine (0.6 mg once or twice daily) is effective for both treatment and prophylaxis. A loading dose of 1.2 mg followed by 0.6 mg one hour later can be used for acute attacks, though this may increase gastrointestinal side effects. Dose adjustment is necessary for renal impairment.

• **Corticosteroids:** Intra-articular injections (triamcinolone 40 mg or methylprednisolone 40–80 mg) are highly effective for monoarthritis with response typically within 24–48 h. Systemic corticosteroids (oral prednisone 20–30 mg daily with tapering over 2–3 weeks) are used for polyarticular attacks or when other options are contraindicated.^[25]^

• **Joint Aspiration:** Therapeutic aspiration of large effusions can provide immediate symptom relief and allows for crystal analysis.

### Chronic Inflammatory Arthritis

For patients with frequent recurrent attacks (≥3 per year) or chronic inflammation, long-term prophylactic therapy is warranted:

• **Low-dose colchicine** (0.5–0.6 mg daily) is the first-line prophylactic agent, reducing attack frequency by approximately 50%.

• **Low-dose corticosteroids** (prednisone ≤5 mg daily) can be used if colchicine is ineffective or not tolerated.

• **Methotrexate** (7.5–15 mg weekly) and Hydroxychloroquine (200–400 mg daily) have been used off-label with some success in refractory cases.^[25,26]^

### Biologic Therapies

For severe, refractory CPPD, biologic agents targeting the IL-1 pathway have shown promise. The rationale for IL-1 inhibition stems from the central role of IL-1β in CPPD pathogenesis: CPP crystals activate the NLRP3 inflammasome in macrophages, leading to caspase-1 activation and subsequent IL-1β release, which drives the acute and chronic inflammatory response.^[1]^ Blocking this pathway addresses the core inflammatory mechanism of the disease. Case series and small studies report good efficacy for anakinra (100 mg subcutaneously daily) and canakinumab (150 mg subcutaneously every 8 weeks) in controlling both acute and chronic inflammation.^[27,28]^ Response rates approach 70%–80%, though treatment is limited by cost and potential side effects.

### Non-pharmacological Management and Surgical Options

**Physical Therapy:** Essential for maintaining range of motion and strength, particularly after acute episodes. Gentle range-of-motion exercises during the acute phase, progressing to strengthening exercises as inflammation resolves.

**Lifestyle Modifications:** Weight management, joint protection strategies, and activity modification can help reduce the frequency of attacks.

**Surgical Intervention:** Reserved for cases with: a) Massive irreparable rotator cuff tears with arthropathy (reverse shoulder arthroplasty); b) Severe joint destruction unresponsive to medical therapy; and c) Large tophaceous deposits causing mechanical symptoms.

### Monitoring and Prognosis

Patients with shoulder CPPD should be monitored for: (1) Attack frequency and severity, (2) Development of chronic arthropathy, (3) Medication side effects, (4) Associated metabolic conditions.

The prognosis is generally favorable with appropriate treatment, though approximately 25% of patients develop chronic arthropathy. Factors associated with poorer prognosis include younger age at onset, polyarticular involvement, and presence of metabolic disorders.

### Management and Follow-up

After diagnosis, the patient was treated with a short course of NSAIDs, followed by low-dose colchicine to prevent recurrent flares. Symptoms improved progressively, with near-complete shoulder recovery by the 6-week follow-up. No further acute episodes occurred over the following months. This favorable evolution supports the diagnosis and illustrates the typically self-limited nature of acute CPP crystal arthritis of the shoulder.

## Conclusion

CPPD of the shoulder is a common but frequently overlooked cause of shoulder pain and dysfunction in the elderly population. The clinical presentation is highly variable, ranging from asymptomatic chondrocalcinosis to destructive arthropathy. Diagnosis can be challenging, often requiring a combination of clinical suspicion, advanced imaging techniques, and, when possible, synovial fluid analysis. The new 2023 ACR/ EULAR classification criteria provide a valuable tool for standardizing diagnosis, particularly in atypical cases where synovial fluid cannot be obtained.

**Key clinical pearls for managing shoulder CPPD include: (**1) maintaining a high index of suspicion in elderly patients with acute shoulder pain, (2) utilizing ultrasound as a readily available diagnostic tool, (3) considering DECT for challenging diagnostic cases, (4) implementing prompt anti-inflammatory therapy for acute attacks, and (5) initiating prophylactic therapy for recurrent disease.

Treatment focuses on managing inflammation, with NSAIDs, colchicine, and corticosteroids forming the cornerstone of therapy. IL-1 inhibitors represent a promising option for refractory disease, though cost remains a limiting factor.

Greater awareness and a systematic diagnostic approach are needed to improve outcomes for patients with this prevalent condition. Future research should focus on prospective studies to better define the natural history of shoulder CPPD, the development of therapies that prevent or dissolve CPP crystal formation, and the validation of imaging-based diagnostic criteria in diverse populations.
